# Differential Contribution of Transcription Factors to *Arabidopsis thaliana* Defense Against *Spodoptera littoralis*

**DOI:** 10.3389/fpls.2013.00013

**Published:** 2013-02-04

**Authors:** Fabian Schweizer, Natacha Bodenhausen, Steve Lassueur, Frédéric G. Masclaux, Philippe Reymond

**Affiliations:** ^1^Department of Plant Molecular Biology, University of LausanneLausanne, Switzerland

**Keywords:** *Arabidopsis thaliana*, *Spodoptera littoralis*, transcription factors, defense, MYC2, MYC3, MYC4

## Abstract

In response to insect herbivory, *Arabidopsis* plants activate the synthesis of the phytohormone jasmonate-isoleucine, which binds to a complex consisting of the receptor COI1 and JAZ repressors. Upon proteasome-mediated JAZ degradation, basic helix-loop-helix transcription factors (TFs) MYC2, MYC3, and MYC4 become activated and this results in the expression of defense genes. Although the jasmonate (JA) pathway is known to be essential for the massive transcriptional reprogramming that follows herbivory, there is however little information on other TFs that are required for defense against herbivores and whether they contribute significantly to JA-dependent defense gene expression. By transcriptome profiling, we identified 41 TFs that were induced in response to herbivory by the generalist *Spodoptera littoralis*. Among them, nine genes, including *WRKY18*, *WRKY40*, *ANAC019*, *ANAC055*, *ZAT10*, *ZAT12*, *AZF2*, *ERF13*, and *RRTF1*, were found to play a significant role in resistance to *S. littoralis* herbivory. Compared to the triple mutant *myc234* that is as sensitive as *coi1-1* to herbivory, knockout lines of these nine TFs were only partially more sensitive to *S. littoralis* but, however, some displayed distinct gene expression changes at the whole-genome level. Data thus reveal that MYC2, MYC3, and MYC4 are master regulators of *Arabidopsis* resistance to a generalist herbivore and identify new genes involved in insect defense.

## Introduction

During million years of coexistence, plants and insects have evolved different types of interactions. Some relationships like pollination are mutually beneficial, whereas the more common predator-host relationship is highly detrimental to plants (Walling, 2000). As a consequence, plants have developed several defense mechanisms to cope with insect attacks including physical barriers, the production of anti-digestive proteins, or toxic secondary metabolites (Howe and Jander, 2007). Most of these defenses are constitutive but are also highly inducible to minimize the cost of triggering defense in times of peace. In *Arabidopsis* and more generally in the Brassicaceae, the amino-acid derived glucosinolates (GS) have been extensively studied for their insect repellent/deterrent properties (Wittstock and Gershenzon, 2002; Halkier and Gershenzon, 2006). These compounds are generally stored as inactive molecules in the vacuole. Upon tissue or cell disruption, GS are catalyzed by myrosinases into active, highly toxic compounds including isothiocyanates, nitriles, and thiocyanates (Grubb and Abel, 2006; Halkier and Gershenzon, 2006).

Several studies have revealed that the plant hormone jasmonate (JA) is the main signal responsible for the activation of inducible defenses against arthropods and necrotrophic fungi (reviewed in Howe and Jander, 2007). In plants, herbivory triggers a burst of JA which leads to a massive transcriptional reprogramming and expression of defense genes (Reymond et al., 2000, 2004; Halitschke et al., 2001; de Vos et al., 2005; Devoto et al., 2005). The F-box protein COI1 was identified as a major component of the JA-pathway, as *coi1-1* mutants were not responding to JA treatment (Xie et al., 1998) and were impaired in the expression of most JA- and insect-inducible genes, including glucosinolate biosynthesis-genes (Reymond et al., 2004; Devoto et al., 2005; Mewis et al., 2006). Consequently, laboratory and field studies have shown that mutants compromised in JA biosynthesis or perception are highly affected in resistance against a wide range of insect herbivores (Howe et al., 1996; McConn et al., 1997; Baldwin, 1998; Stintzi et al., 2001; Li et al., 2004; Reymond et al., 2004; Paschold et al., 2007).

For years, the precise mode of JA perception had remained elusive until several studies provided evidence that COI1 itself, together with members of the JAZ family of repressors, forms a complex with jasmonate-isoleucine (JA-Ile), an amino-acid conjugated form of JA (Chini et al., 2007; Thines et al., 2007; Yan et al., 2007). Further work demonstrated that (+)-7-*iso*-Jasmonoyl-l-isoleucine is the natural and bioactive ligand of COI1-JAZ complexes (Fonseca et al., 2009). In the absence of JA-Ile, reflecting the state of non-attacked plants, JAZ proteins interact with the bHLH MYC2 transcription factor (TF) and NINJA, which in turn interacts with TPL to actively repress transcription of MYC2 target genes (TG; Pauwels et al., 2010). Upon herbivory, the accumulation and binding of JA-Ile to COI1 leads to ubiquitination and subsequent degradation of JAZs via the 26S proteasome, allowing MYC2 to activate the expression of JA-responsive genes (Sheard et al., 2010; Pauwels and Goossens, 2011).

While mechanisms of JA perception are being unveiled, relatively little is known on which transcription factors (TFs) are controlling such a massive transcriptional reprogramming and on which downstream genes are important for defense against herbivores. Although MYC2 has been shown to interact with JAZs and therefore potentially activate JA-responsive genes, several studies reported that contrary to *coi1-1* that is male sterile, *myc2* alleles are fully fertile; moreover, they are only partially sensitive to exogenous JA and are only slightly more susceptible to insect herbivory than wild-type plants (Boter et al., 2004; Lorenzo et al., 2004; Dombrecht et al., 2007; Fernández-Calvo et al., 2011; Verhage et al., 2011). Recently, MYC2 was found to act additively with its closely related homologs MYC3 and MYC4 to control JA responses, including defense against herbivory (Fernández-Calvo et al., 2011). Indeed, a triple mutant *myc2myc3myc4* (*myc234*) was as susceptible as *coi1-1* to the generalist herbivore *Spodoptera littoralis* and had a similar reduced expression of JA marker genes (Fernández-Calvo et al., 2011). Besides MYC factors, the insect-inducible *Arabidopsis* MYB102 was found to be necessary for defense against the specialist *Pieris rapae* (de Vos et al., 2006). A*myb102* mutant showed lower expression of defense- and cell wall-related genes. However, its connection with the JA-pathway was not examined (de Vos et al., 2006). Enhanced expression of *MYB75* (*PAP1*), a gene that controls phenylpropanoid metabolism, by activation-tagging in *Arabidopsis* slowed growth of *Spodoptera frugiperda*, but the molecular mechanism of this response was not investigated (Johnson and Dowd, 2004). Similarly, heterologous expression of MYB12 in tobacco conferred increased resistance to *Spodoptera litura* and *Helicoverpa armigera*, presumably by the enhanced accumulation of flavonoids (Misra et al., 2010). Two WRKY TFs from *Nicotiana attenuata*, WRK3 and WRK6, were found to positively control the accumulation of JA-Ile and susceptibility to *Manduca sexta*, suggesting that these factors play a role upstream of the JA-pathway (Skibbe et al., 2008). Finally, GS biosynthesis is regulated by six R2R3-MYB TFs. MYB28, MYB29, and MYB76 control aliphatic-GS genes (Hirai et al., 2007; Gigolashvili et al., 2008; Sønderby et al., 2010), whereas MYB34, MYB51, and MYB122 control indole-GS genes (Gigolashvili et al., 2007). Overexpression of MYB51 in *Arabidopsis* impaired growth of *Spodoptera exigua* (Gigolashvili et al., 2007) whereas a *myb28myb29* double mutant lacking aliphatic-GS was more susceptible to feeding by *Mamestra brassicae* (Beekwilder et al., 2008).

To identify novel TFs that respond to herbivory and to gain insight on their relative contribution to defense, we carried-out a transcriptomic search of insect-inducible TFs. We found nine TFs that had a significant effect on insect performance and analyzed insect-induced transcriptome changes in respective knockout lines. Our study reveals new players in *Arabidopsis* defense against a generalist herbivore and highlights the predominant role of MYC2, MYC3, and MYC4.

## Materials and Methods

### Plant material and growth conditions

*Arabidopsis thaliana* Col-0 was the genetic background of all mutant lines used in this study. The following T-DNA insertion lines were obtained from the Nottingham *Arabidopsis* Stock Center: *erf13* (GK_121A12), *nac019* (Salk_096295), *nac055* (SALK_014331), *wrky18* (SALK_093916), *zat10* (SALK_054092), *zat12* (SAIL_347_G03), *azf2-1* (SALK_132562), *rap2.6* (SAIL_1225_G09), *rrtf1* (SALK_150614), *myb44* (SALK_039074). Homozygous lines were selected by PCR and absence of transcription of the TG in mutant lines was confirmed by RT-PCR. Specific forward and reverse primers were designed with SIGnAL T-DNA verification tool for all lines^1^. We generated *nac019nac055* by crossing single mutants. Seeds of the triple mutant *myc2myc3myc4* were a gift from Roberto Solano (Centro Nacional de Biotecnología-CSIC, Madrid, Spain). The *coi1-1* (non-glabrous) mutant was obtained from Jane Glazebrook (University of Minnesota, St. Paul, MN, USA) and *wrky40* and *wrky18wrky40* mutants were obtained from Imre Somssich (Department of Plant Microbe Interactions, Max Planck-Institute for Breeding Research, Cologne, Germany).

Col-0 and mutant lines were stratified in water for 4 days at 4°C. The *myc2myc3myc4* mutant was stratified in water containing 0.1 mM gibberellic acid to stimulate germination. Seeds were then transferred to pots containing potting compost. The *coi1-1* mutant was germinated on Murashige and Skoog medium (Sigma, Buchs, Switzerland) containing 3% sucrose and 30 μM JA and incubated under continuous light (150 μmol m^−2^ s^−1^) for 7 days in a growth chamber. Homozygous *coi1-1* mutants showing normal greening of leaves and no inhibition of root growth (Feys et al., 1994) were transferred to pots. Plants were grown in a growth chamber as previously described (Reymond et al., 2000).

### Insect bioassays

*Spodoptera littoralis* (Egyptian cotton worm) eggs were obtained from Syngenta (Stein, Switzerland) and were stored at 10°C until further use. Eggs were placed in a beaker covered with plastic film in an incubator (26°C) for 2–3 days to allow hatching. Larvae were then reared on *Arabidopsis* plants. For initial insect challenge, two to three fourth- or fifth-instar *S. littoralis* larvae were allowed to feed on 6-week-old plants for 4–5 h in a transparent plastic box in a growth chamber (20°C, 65% relative humidity, 100 μmol m^−2^ s^−1^, 10/14 h photoperiod) until approximately 20% of leaf area was removed. For each experiment, damaged leaf tissue from 12 challenged plants was harvested and immediately stored in liquid nitrogen. Leaves from 12 control, unchallenged plants were collected at the same time. For longer feeding experiments, newly hatched larvae (three for two plants) were allowed to feed continuously during 8 days until leaves were harvested. Microarray analyses with Col-0 and *coi1-1* plants were performed on at least three independent biological replicates.

For testing the susceptibility of TF mutants, 3-week-old plants were used. Forty newly hatched *S. littoralis* larvae were placed in a transparent plastic box containing 70 plants. After 8 days of feeding, larvae were collected and weighed on a precision balance (Mettler-Toledo, Greifensee, Switzerland) whereas plant tissues from control and treated plants were immediately stored in liquid nitrogen and used for microarray analyses. All experiments were repeated at least three times independently, except for *erf13* and *rrtf1* mutants (two replicates).

### Microarray experiments and data analysis

For microarray analysis, total RNA of plant tissues was extracted, reverse-transcribed, and processed according to a previously published procedure (Bodenhausen and Reymond, 2007). Labeled probes were hybridized onto CATMAv4 microarrays containing 32,998 *Arabidopsis* gene-specific tags and gene-family tags (Sclep et al., 2007). Hybridization and scanning have been described previously (Reymond et al., 2004). Data normalization and statistical analyses including false-discovery rate (FDR) correction were carried-out using an interface developed at the University of Lausanne [Gene Expression Data Analysis Interface (GEDAI; Liechti et al., 2010)]. Hierarchical clustering of microarray data as well as gene node heights calculations were done with Multi experiment viewer software^2^ using the default options. Microarray data have been submitted to ArrayExpress database under accession E-MTAB-1418^3^.

### Quantitative RT-PCR

Leaf samples from 5 to 10 plants were harvested and pooled after 48 h of herbivory by first-instar *S. littoralis* larvae. Tissue samples were ground in liquid nitrogen and total RNA was extracted using RNeasy Plant Mini Kit and treated with DNaseI (Qiagen, Hombrechtikon, Switzerland). Afterward, cDNA was synthesized from 1 μg of RNA using M-MLV reverse transcriptase (Invitrogen, Zug, Switzerland) in a final volume of 25 μl and subsequently diluted fourfold with water. Gene-specific primers were designed to produce amplicons between 80 and 120 bp. Primer efficiencies (E) were evaluated by five-step dilution regression. Quantitative real-time PCR (qRT-PCR) was performed using Brilliant II Fast SYBR-Green qRT-PCR Master Mix. Reactions were done in a final volume of 25 μl containing 12.5 μl of 2× SYBR, 3.75 μl of ROX (1/5000 dilution), 4.25 μl of RNAse-free water, 2.5 μl of primer mix (each primer at 1 μM), and 2 μl of cDNA. A Mx3000P real-time PCR instrument (Agilent, Morges, Switzerland) was used with the following program: 95°C for 5 min, then 40 cycles of 10 s at 95°C, 20 s at 55°C, and 30 s at 60°C. Values were normalized to the house-keeping gene *ACTIN8*. The expression level of a TG was normalized to the reference gene (RG) and calculated as Normalized Relative Quantity (NRQ) as follows: NRQ = *E*^CtRG^/*E*^CtTG^. Each experiment was repeated three times independently.

### Glucosinolate analysis

For GS extraction, seven 3-week-old plants were challenged for 48 h with two neonate *S. littoralis* larvae per leaf. Unchallenged plants were used as controls. Samples from four biologically independent replicates were analyzed. Extraction method, UHPLC-QTOFMS measurements and analysis have been recently described (Glauser et al., 2012).

## Results

### Identification of insect-induced transcription factors

**Figure 1:**
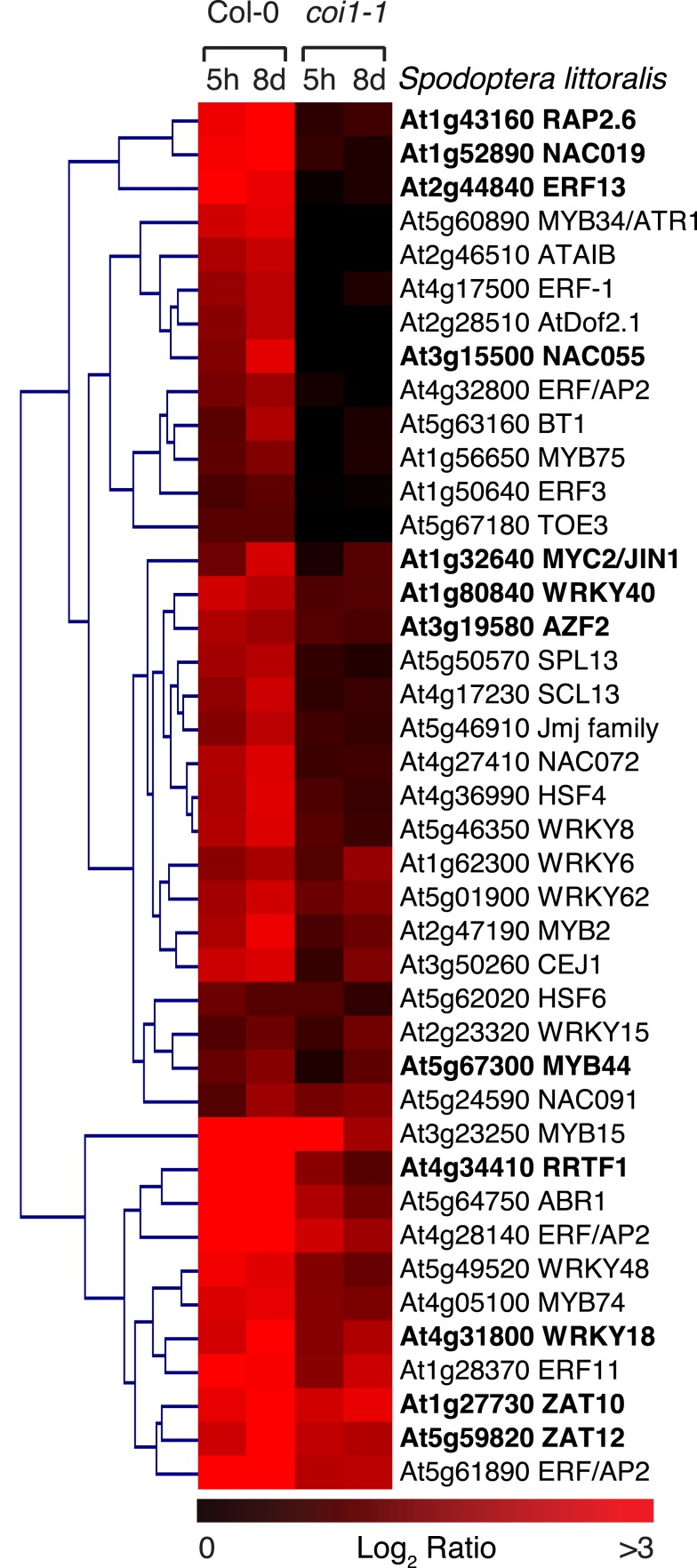
**Expression of insect-inducible transcription factors in wild-type and *coi1-1***. Heat map representing transcription factors induced in response to *Spodoptera littoralis* in Col-0 and their expression in the *coi1-1* mutant. Plants were challenged for 5 h with fourth–fifth instar larvae or for 8 days with first-instar larvae. Genes significantly induced (log_2_ ratio > 1, *P*-value < 0.05) were represented in a clustered heat map with MultiExperiment Viewer 4.8.1 using Euclidian distance. Genes in bold were analyzed in this study.

To identify novel TFs that are involved in the response to herbivory, we reasoned that some of these factors might be themselves subjected to transcriptional regulation. We therefore performed a whole-genome microarray analysis of *Arabidopsis* plants challenged with the generalist *S. littoralis* and searched for TF genes that were robustly induced by herbivory. We collected RNA from several independent replicates after 5 h of feeding with fourth–fifth instar larvae and after 8 days of feeding with neonate larvae and analyzed the transcriptome using *Arabidopsis* CATMA microarrays (Sclep et al., 2007). In addition, to evaluate the role of the JA-pathway in regulating these TFs, we used *coi1-1* plants in the same experimental set-up. Induced genes were defined as genes with a mean expression ratio ≥ 2 in Col-0 (adjusted *P*-value < 0.05). Based on TAIR annotation^4^, we identified 41 TFs that were significantly up-regulated by *S. littoralis* herbivory (Table S1 in Supplementary Material). Clustering microarray data of Col-0 and *coi1-1* plants showed that most TFs were not or much less induced in *coi1-1* plants, suggesting that they depend on a functional JA-pathway (Figure 1; Table S1 in Supplementary Material). Induced TFs belonged to different classes, including for example several ERF/AP2, bHLH, MYB, WRKY, Zinc-fingers, and NAC factors.

### Insect performance on TF knockout lines

**Figure 2:**
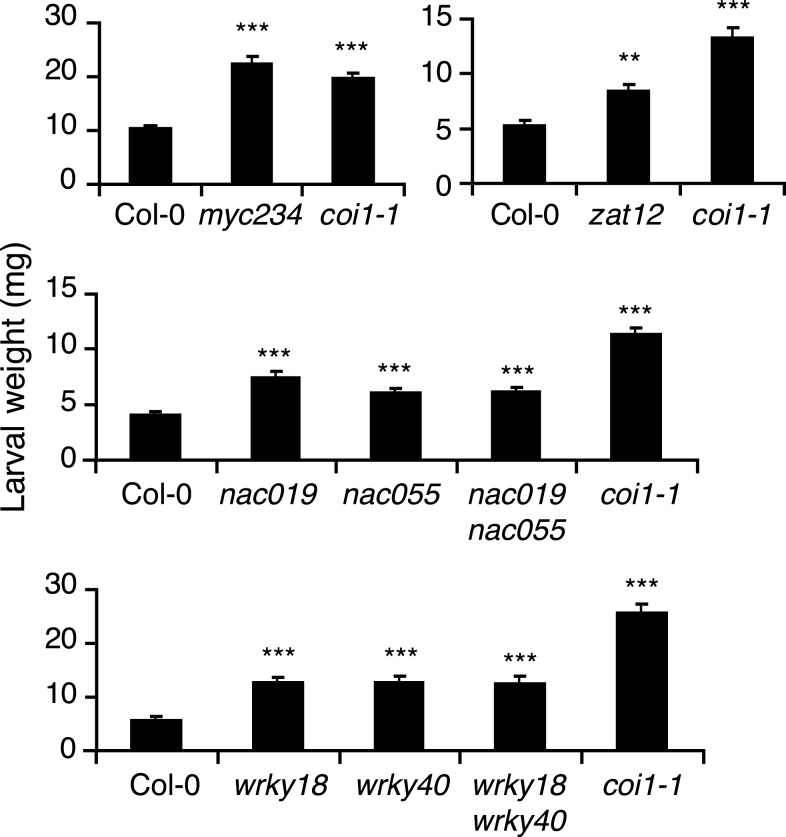
**Insect performance on transcription factor mutants**. Freshly hatched *S. littoralis* larvae were placed on each genotype and larval weight (mean ± SE) was measured after 8 days of feeding. Asterisks indicate statistically significant differences between mutant plants and Col-0 (Student’s *t*-test, *P < 0.05, **P < 0.01, ***P < 0.001). Similar results were observed in at least three independent replicate experiments.

Larval growth can be used as an outcome of plant defense ability against herbivores. To assess whether the newly identified insect-induced TFs where involved in defense, we obtained T-DNA knockout lines and challenged them with insects. For this assay, 3-week-old plants were subjected to feeding by neonate *S. littoralis* for 8 days. Among the 41 insect-induced TFs, some were already known to be involved in defense against herbivory (MYC2, MYB34, MYB75) and were not tested further. For the other candidates, we obtained 11 homozygous mutant lines, of which nine showed a significantly higher growth of *S. littoralis* larvae (Figure 2; Table 1). Larval weight was between 27% (*erf13*) and 66% (*zat12*) higher on mutant than on wild-type plants, but this was less pronounced than on *coi1-1* or *myc234* plants (>300%; Table 1). Interestingly, all sensitive TF mutants belonged to unrelated gene families like bHLH (*myc234*), WRKY (*wrky18*, *wrky40*), NAC (*nac019*, *nac055*), zinc-finger (*zat10*, *zat12*, *azf2-1*), and ERF/AP2 (*erf13* and *rrtf1*). For some closely related TFs like WRKY18, WRKY40, and NAC019, NAC055, the respective double mutants were also tested. Noteworthy, although both single mutants were significantly more sensitive to herbivory, none of the double mutants showed an additive effect on larval growth (Figure 2). A plausible explanation could be that these factors form heterodimers and control the same sets of defense genes.

**Table 1:** **Insect performance on transcription factor mutants**.

Mutant	AGI	Relative weight
*coi1-1*	At2g39940	3.00 ± 0.23***
*myc234*	At1g32640/At5g46760/At4g17880	3.10 ± 0.42***
*nac019*	At1g52890	1.50 ± 0.14***
*nac055*	At3g15500	1.38 ± 0.17***
*nac019nac055*	At1g52890/At3g15500	1.40 ± 0.24***
*zat10*	At1g27730	1.45 ± 0.28***
*zat12*	At5g59820	1.66 ± 0.05***
*azf2-1*	At3g19580	1.54 ± 0.07***
*wrky18*	At4g31800	1.57 ± 0.20***
*wrky40*	At1g80840	1.54 ± 0.38**
*wrky18wrky40*	At4g31800/At1g80840	1.46 ± 0.13***
*rrtf1-1*	At4g34410	1.30 ± 0.08***
*erf13*	At2g44840	1.27 ± 0.13***
*rap2.6*	At1g43160	1.32 ± 0.22 n.s.
*myb44*	At5g67300	1.00 ± 0.11 n.s.

Relative weight corresponds to the mean weight of neonate *S. littoralis* larvae feeding on 3-week-old mutants for 8 days divided by the mean weight of larvae feeding on Col-0. Values (±SE) are the mean of several replicates (mutants *n* ≥ 3; *myb44n* = 2; Col-0 *n* = 17). Asterisks indicate *P*-value (n.s. not significant; * < 0.05; ** < 0.01; *** < 0.001; Nested ANOVA).

### Expression of JA marker genes

**Figure 3:**
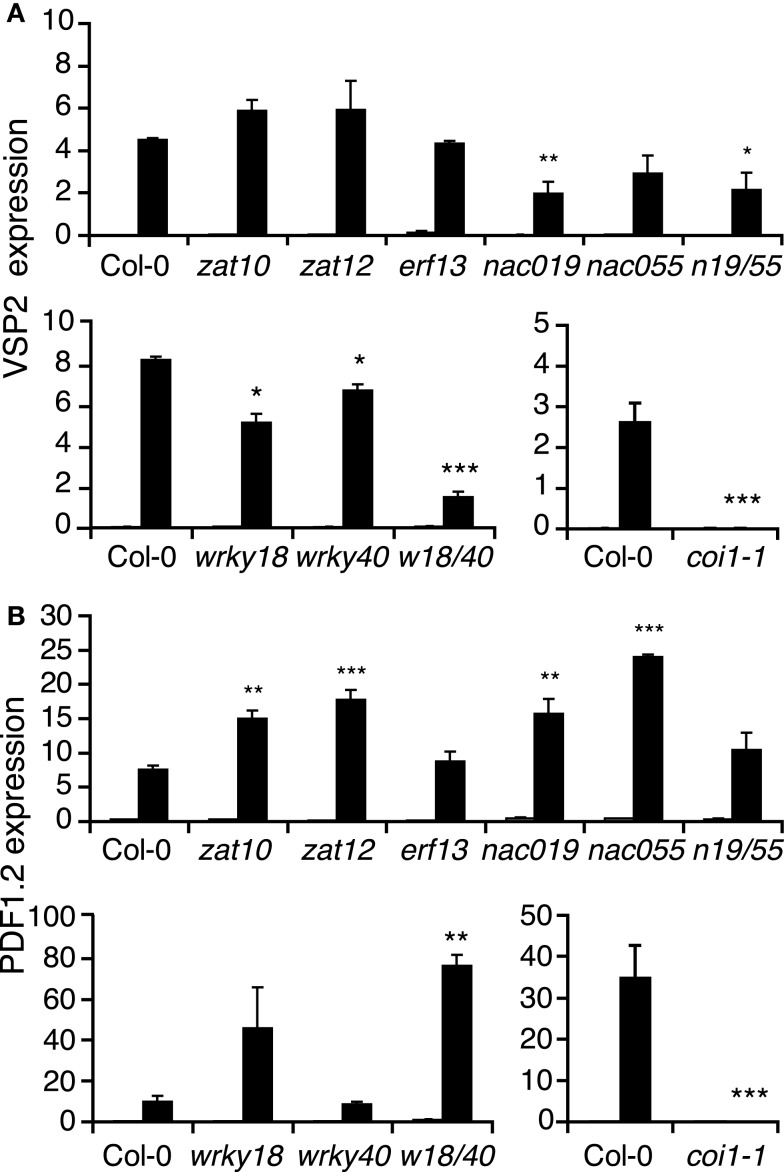
**Expression of jasmonate marker genes in transcription factor mutants**. Relative expression of *VSP2* **(A)** and *PDF1.2* **(B)** was measured by qRT-PCR in untreated plants (white bars) and in plants challenged for 48 h with *S. littoralis* larvae (black bars). Values are the mean ± SE of three biological replicates. Asterisks indicate statistically significant differences in treated mutant plants compared to treated Col-0 plants (Student’s *t*-test, *P < 0.05, **P < 0.01).

Several studies have shown that the JA-pathway positively controls the expression of at least two distinct sets of genes. Herbivory leads to a burst of JA which activates the expression of genes like *JAZ10* and *VSP2* (Reymond et al., 2004; Yan et al., 2007). In response to necrotrophic fungi, plants produce JA and ethylene (ET), which together turn on a set of genes including *PDF1.2* and *ORA59* (Manners et al., 1998; Penninckx et al., 1998; Pré et al., 2008). To test the involvement of insect-responsive TFs in the activation of these two branches of the JA-pathway, we monitored *VSP2* and *PDF1*.2 expression in mutant lines by qRT-PCR. *VSP2* induction by *S. littoralis* was significantly reduced in *nac019*, *nac019nac055*, *wrky18*, *wrky40*, *wrky18wrky40* mutants, although to a lesser extent than in *coi1-1*, but was not affected in *nac055*, *zat10*, *zat12*, and *erf13* mutants (Figure 3). Interestingly, up-regulation of *PDF1.2* was higher in all *nac* mutants, as well as in *wrky18*, *wrky18wrky40*, *zat10*, and *zat12*, than in Col-0. On the contrary, *PDF1.2* expression was abolished in *coi1-1* (Figure 3). Noteworthy, such opposite expression of *PDF1.2* was previously observed between *myc234* and *coi1-1* in response to JA treatment (Fernández-Calvo et al., 2011). Our results suggest that increased insect susceptibility of some TF mutant lines can be explained by a reduced activation of the JA-pathway that leads to the accumulation of anti-insect proteins, including VSP2.

### Whole-genome analysis of TF mutants

**Figure 4:**
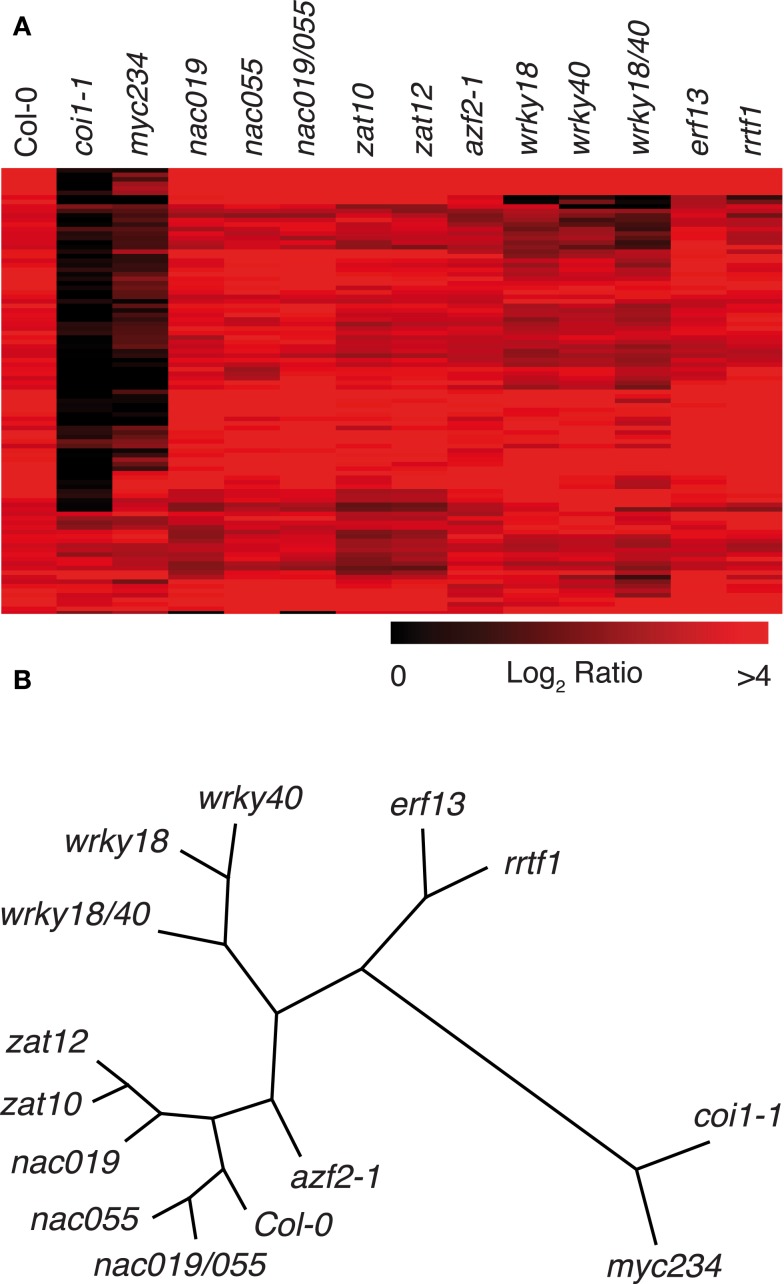
**Whole-genome expression profile of transcription factor mutants. (A)** Heat map clustering the 100 most highly induced genes in Col-0 plants after 8 days of insect feeding and their respective expression in mutant plants. Heat map was created with MultiExperiment Viewer 4.8.1. **(B)** Correspondence analysis of expression profiles including all insect-induced genes (log_2_ ratio > 1, *P*-value < 0.05; *n* = 874). Clustering and node length calculations were performed with MultiExperiment Viewer 4.8.1 and represented as unrooted tree in Treeview 1.6.6 (http://taxonomy.zoology.gla.ac.uk/rod/treeview.html).

To gain more insight on the role of insect-induced TFs on downstream gene expression, we carried-out microarray analyses with mutant lines that showed a higher sensitivity to *S. littoralis*. As controls for highly sensitive mutants, we included *coi1-1* and *myc234*. *S. littoralis* larvae were allowed to feed for 8 days on Col-0 and mutant plants, then RNA was extracted and hybridized to CATMA microarrays. As expected, the majority of genes induced by herbivory in Col-0 were JA-dependent and thereby were not induced in *coi1-1* (Figure 4A). In accordance with their similar insect susceptibility, *myc234* and *coi1-1* showed a very similar expression profile, corroborating the additive role of MYC2, MYC3, and MYC4 as general transcriptional regulators acting directly downstream of COI1 to control the expression of JA-responsive genes (Fernández-Calvo et al., 2011).

Although other TF mutants showed an overall expression pattern that was more similar to Col-0, they anyhow displayed altered profiles (Figure 4A). A correspondence analysis where the weight distance between different experiments is indicative of their relative similarity indicated that *coi1-1* and *myc234* expression profiles form a distinct subgroup that is distant from a second subgroup containing Col-0 and all TF mutant profiles (Figure 4B). In this second subgroup, *wrky18* and *wrky 40* mutants formed a clearly separated branch, as well as *erf13* and *rrtf1*, two members of the B3 sub-family of ERF/AP2 TFs that clustered together, whereas *nac019*, *nac055*, *zat10*, *zat12*, and *azf2-1* were more similar to Col-0 (Figure 4B).

Although *coi1-1* and *myc234* expression profiles were globally similar, we could however detect significant differences in the expression of several genes. We observed that some COI1-dependent genes were normally expressed in *myc234*, as for instance *PDF1.2* and a myrosinase-associated protein (At1g54010), or showed a reduced induction, as for instance *VSP2*, *CORI3*, and *MYB75* (Table 2; Table S2 in Supplementary Material). Thus, the distance separating transcriptomes of *coi1-1* and *myc234* on the cluster (Figure 4B) probably reflects the expression changes of such genes.

**Table 2:** **List of insect-induced genes**.

		Expression ratio (log_2_)		
Description	AGI	Col-0	*coi1-1*	*myc234*
TI1, trypsin inhibitor	At2g43510	4.79***	3.32**	4.07**
RD20, calcium-binding protein	At2g33380	4.57***	3.33**	3.74**
Protease inhibitor (LTP)	At4g12500	4.27**	4.49**	5.03**
Aldo/keto reductase	At2g37770	4.17***	3.78***	3.51**
CAD8, cinnamyl-alcohol dehydrogenase	At4g37990	3.86**	3.26**	3.10**
Strictosidine synthase	At1g74010	3.64**	3.74*	3.36**
Protease inhibitor (LTP)	At4g12490	3.63**	3.67**	4.03**
Protease inhibitor	At2g38870	3.55**	3.48**	3.72**
PRX52, peroxidase	At5g05340	3.53**	4.17**	4.14**
Trypsin and protease inhibitor	At1g73260	3.51**	2.20*	2.74*
FAD-binding berberine family protein	At4g20860	3.59***	2.26*	2.63**
FAD-binding berberine family protein	At2g34810	3.82***	1.77*	2.04*
PDF1.2, plant defensin	At5g44420	4.33*	0.23	4.09**
Myrosinase-associated protein	At1g54010	4.20**	0.77	3.67**
Oxidoreductase, 2OG-Fe(II) oxygenase	At5g05600	4.57***	0.03	3.50**
GCN5-related N-acetyltransferase (GNAT)	At2g39030	5.57***	−0.34	2.78*
Palmitoyl protein thioesterase	At4g17470	5.25***	−0.05	2.54**
VSP2, acid phosphatase	At5g24770	5.03***	0.81	2.52**
CORI3, cystine lyase	At4g23600	4.16***	0.55	2.15*
BAM5, beta-amylase	At4g15210	3.84***	−0.54	2.44*
PAP1 (MYB75), transcription factor	At1g56650	3.75**	0.58	2.72*
Terpene synthase/cyclase,	At1g61120	4.84***	0.07	1.23*
Oxidoreductase, 2OG-Fe(II) oxygenase	At2g38240	4.54**	−0.24	1.36*
Jacalin lectin	At1g52000	4.42**	−0.39	1.54*
TSA1, calcium-binding protein	At1g52410	4.13**	0.41	1.78**
SSRP1, DNA-binding protein	At3g28730	4.10***	0.77	1.32*
GOLS1, galactinol synthase	At2g47180	4.08***	0.85	1.36*
Expressed protein	At4g02360	4.02***	0.60	1.69
ARGAH2, arginase	At4g08870	3.96**	−0.15	1.54*
DHAR1, dehydroascorbate reductase	At1g19570	3.89***	0.11	1.42*
Cysteine proteinase	At4g11320	3.79***	−0.06	1.54**
ILL6, IAA amino-acid conjugate hydrolase	At1g44350	3.59**	−0.56	1.51*
AOC1, allene oxide cyclase	At3g25760	3.58**	−0.18	1.26*
LOX3, lipoxygenase	At1g17420	3.97**	0.87	0.96*
Protein kinase	At4g10390	3.68**	0.21	0.93*
Oxidoreductase, 2OG-Fe(II) oxygenase	At3g55970	3.90**	0.14	0.99*
jacalin lectin,	At2g39330	4.92***	−0.57	0.99*
FAMT, farnesoic acid methyl transferase	At3g44860	3.79***	−1.17	0.82
VSP1, acid phosphatase	At5g24780	6.48***	0.59	0.47
JAZ10	At5g13220	4.83***	−0.33	0.39
MBP2, myrosinase-binding protein	At1g52030	4.74***	0.33	−0.24
MBP1, myrosinase-binding protein	At1g52040	4.55***	0.39	−0.10
TRAF-like family protein	At5g26260	4.33***	0.40	0.38
UTR3, UDP-galactose transporter	At1g14250	3.86**	−0.42	0.43
Trypsin and protease inhibitor	At1g73325	3.85***	−0.19	0.37
*O*-methyltransferase	At1g76790	3.74***	−1.09	−0.68
TRAF-like family protein	At3g28220	3.72***	−1.12	0.07
AT14A, transmembrane protein	At3g28300	3.70*	−1.06	−1.09
AT14A, transmembrane protein	At3g28290	3.58*	−1.12	−0.60
PGL5, 6-phosphogluconolactonase	At5g24420	3.56***	−0.83	−0.79*

List of the 50 most highly induced genes in response to *S. littoralis* in Col-0. Three-week-old plants were challenged for 8 days with first-instar larvae. Values are calculated from several independent biological replicates (Col-0, *myc234n* = 4; coi1-1: *n* = 3). Ratios are color-coded according to intensity: yellow (from 1 to 2), magenta (from 2 to 3), red (>3). Asterisks indicate adjusted *P*-value (* < 0.05; ** < 0.01; *** < 0.001).

**Figure 5:**
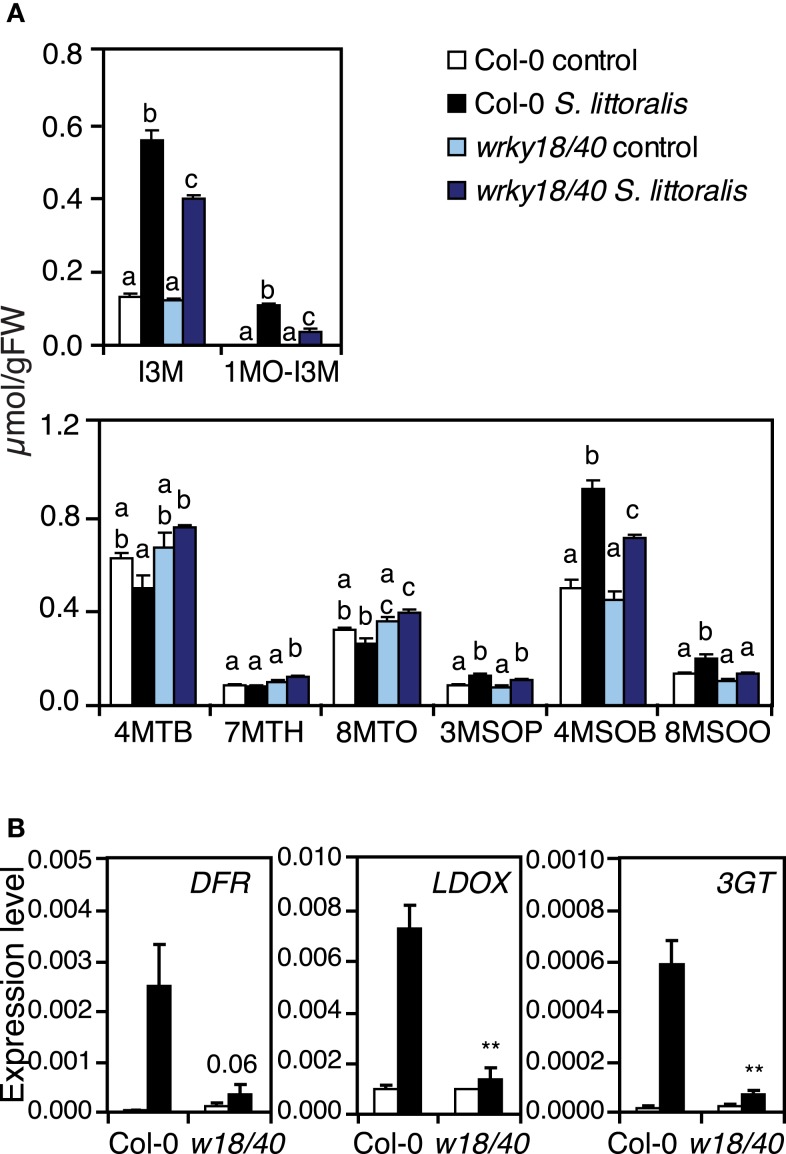
**Quantification of glucosinolates and expression of phenylpropanoid pathway genes in *wrky18wrky40* mutant**. **(A)** Levels of eight glucosinolates were quantified in Col-0 and *wrky18wrky40* double mutant. Plants were challenged for 2 days with *S. littoralis* larvae. Unchallenged plants were used as controls. Values are the mean (±SE) of four biological replicates. Bars with different letters differ at *P* < 0.05 (Tukey’s HSD test). 4MTB, 4-Methylthiobutyl-GS; 7MTH, 7-Methylthioheptyl-GS; 8MTO, 8-Methylthiooctyl-GS; 3MSOP, 3-Methylsulfinylpropyl-GS; 4MSOB, 4-Methylsulfinylbutyl-GS; 8MSOO, 8-Methylsulfinyloctyl-GS; I3M, Indol-3-ylmethyl-GS; 1MO-I3M, 1-Methoxyindol-3-ylmethyl-GS. **(B)** The *wrky18wrky40* mutant shows altered expression of phenylpropanoid pathway genes *DFR*, *LDOX*, and *3GT* in response to herbivory. Relative expression was measured by qRT-PCR in untreated plants (white bars) and in plants challenged for 48 h with *S. littoralis* larvae (black bars). Values are the mean (±SE) of three replicate experiments. Asterisks indicate statistically significant differences in treated *wrky18wrky40* plants compared to treated Col-0 plants (Student’s *t*-test, *P < 0.05, **P < 0.01, ***P < 0.001).

A careful examination of the expression profiles of *nac019*, *nac055*, *nac055nac019*, *zat10*, *zat12*, *azf2-1*, *rrtf1*, and *erf13* mutants did not allow to identify candidate defense genes that could easily explain the susceptibility to *S. littoralis*. As illustrated by the clustering of TF mutant expression profiles with Col-0 (Figure 4B), the large majority of insect-inducible genes were still up-regulated in the mutants (Table S2 in Supplementary Material). However, consistent with the fact that *wrky* mutants formed a distinguishable group in the cluster, they showed a partially reduced expression of genes from several pathways including general defense (protease inhibitors), JA-biosynthesis (LOX2), GS biosynthesis (MYB34, CYP79B3), and breakdown (TGG2), and phenylpropanoid biosynthesis (DFR, CHS; Table S2 in Supplementary Material). To test whether the transcriptional change in GS biosynthesis-genes could effectively alter GS biosynthesis, we quantified GS in *wrky18wrky40* by UHPLC-QTOFMS (Glauser et al., 2012). Analysis of the most abundant GS showed that, in response to *S. littoralis*, *wrky18wrky40* accumulated significantly more methylthio-GS (4MTB, 7MTH, 8MTO), less methylsulfyl-GS (4MSOB, 8MSOO), and less indole-GS (I3M, 1MO-I3M) than Col-0 (Figure 5A). Thus, although the total GS amount between *wrky18wrky40* and Col-0 was similar, these qualitative differences could contribute to the increased insect susceptibility of the mutant.

To test the involvement of WRKY18 and WRKY40 in the phenylpropanoid pathway, we analyzed three genes involved in the last steps of anthocyanins and flavonols biosynthesis. Expression analysis by qRT-PCR showed clearly that DFR, LDOX, and 3GT were strongly induced by herbivory in Col-0, whereas no significant induction could be observed in *wrky18wrky40* (Figure 5). The insect sensitive phenotype of *wrky18wrky40* could therefore be explained in part by a reduced accumulation of metabolites from the phenylpropanoid pathway.

### Expression of insect-inducible TFs in *coi*1-1 and *myc*234

**Figure 6:**
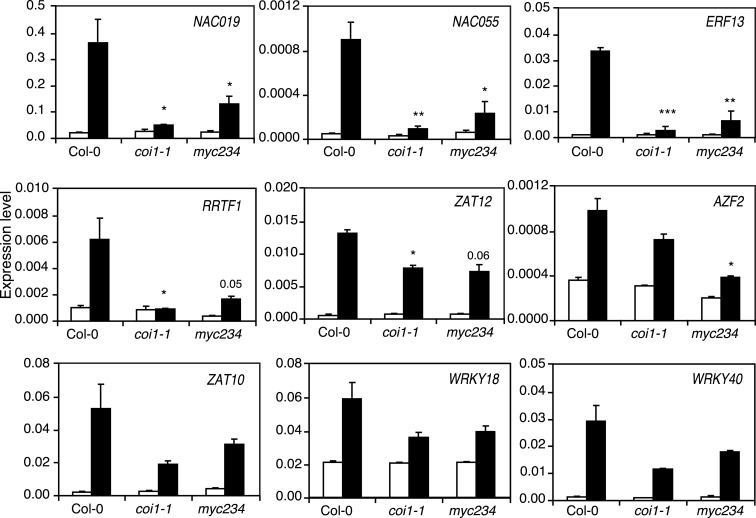
**Expression of insect-inducible transcription factors in *coi1-1* and *myc234***. Expression of TFs was measured by qRT-PCR in untreated plants (white bars) and in plants challenged for 48 h with *S. littoralis* larvae (black bars). Values are the mean (±SE) of three biological replicates. Asterisks indicate statistically significant differences in treated plants compared to Col-0 (Student’s *t*-test, *P < 0.05, **P < 0.01, ***P < 0.001).

We found that some TF mutants show an altered expression of JA marker genes but that this was not as severe as in *coi1-1* and *myc234* plants (Figure 3, Table S2 in Supplementary Material). We thus wondered whether this regulation was done through the COI1/MYC234 signaling module or whether these TFs were independent modulators of defense gene expression. To address this hypothesis, we analyzed the expression of nine TFs whose mutants were more sensitive to insects in Col-0, *coi1-1*, and *myc234*. All tested TFs were highly induced in response to *S. littoralis*, validating the microarray data (Figure 6). Moreover, TF expression pattern in *coi1-1* and *myc234* could be separated into two different types of responses. First, *NAC019*, *NAC055*, *ERF13*, and *RRTF1* were all significantly less induced in *coi1-1* and *myc234* than in Col-0 (Figure 6). Interestingly, *NAC019*, *NAC055*, *ERF13*, and *RRTF1* were barely induced in *coi1-1* mutants but did still show a slight induction in *myc234*. Taken together, it seems that these genes depend on a functional JA-pathway and are thus not induced in *coi1-1*, whereas a redundant MYC or other TFs might contribute to their partial expression in *myc234*. The second group included genes whose expression was still induced in *coi1-1* and *myc234*, but somewhat reduced when compared to Col-0 (Figure 6). Induction of *ZAT10*, *ZAT12*, *AZF2*, *WRKY18*, and WRKY40 was reduced in *coi1-1* and *myc234* compared to Col-0, although the difference with Col-0 was only statistically significant for *ZAT12* in *coi1-1* and *AZF2* in *myc234*.

Previous reports have shown that MYC2, MYC3, and MYC4 bind preferentially to G-box and G-box like sequences in the promoter of TGs (Dombrecht et al., 2007; Fernández-Calvo et al., 2011; Godoy et al., 2011). We further investigated whether there was a correlation between TF expression patterns and the presence of MYC2 binding *cis*-elements in their respective promoters. Nearly all promoters contained G-box and G-box like sequences, indicating that they might be direct targets of MYCs (Table 3). The exception was *WRKY18* and *ERF13* that did not contain any G-box element. Since *ERF13* expression was strongly dependent on COI1 and MYC2, MYC3, MYC4 (Figure 6), this gene must thus be indirectly controlled by MYCs. Taken together, our findings suggest that enhanced insect performance on *coi1-1* and *myc234* is explained in part by a reduced expression of downstream TFs that regulate the expression of defense genes.

**Table 3:** **MYC-binding sites in the promoter of insect-induced TFs**.

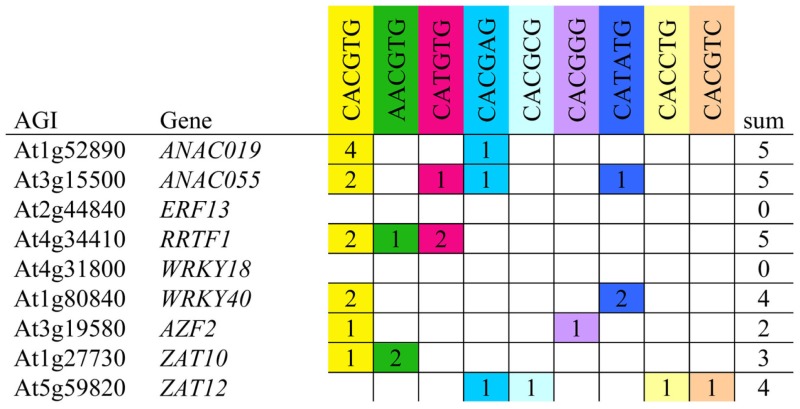

*Number of G-box and G-box like *cis*-elements in 1 *kb5*′-upstream region of TFs (TAIR7_upstream_1000) was identified by using Promomer (http://bar.utoronto.ca/welcome.htm). MYC*-*binding affinities on G-box and G-box like motifs have been previously studied (Godoy et al., 2011). The first sequence from the left represents the canonical G-box with the highest affinity, whereas the eight other sequences represent G-box like motifs with decreasing affinity (Godoy et al., 2011)*.

## Discussion

**Figure 7:**
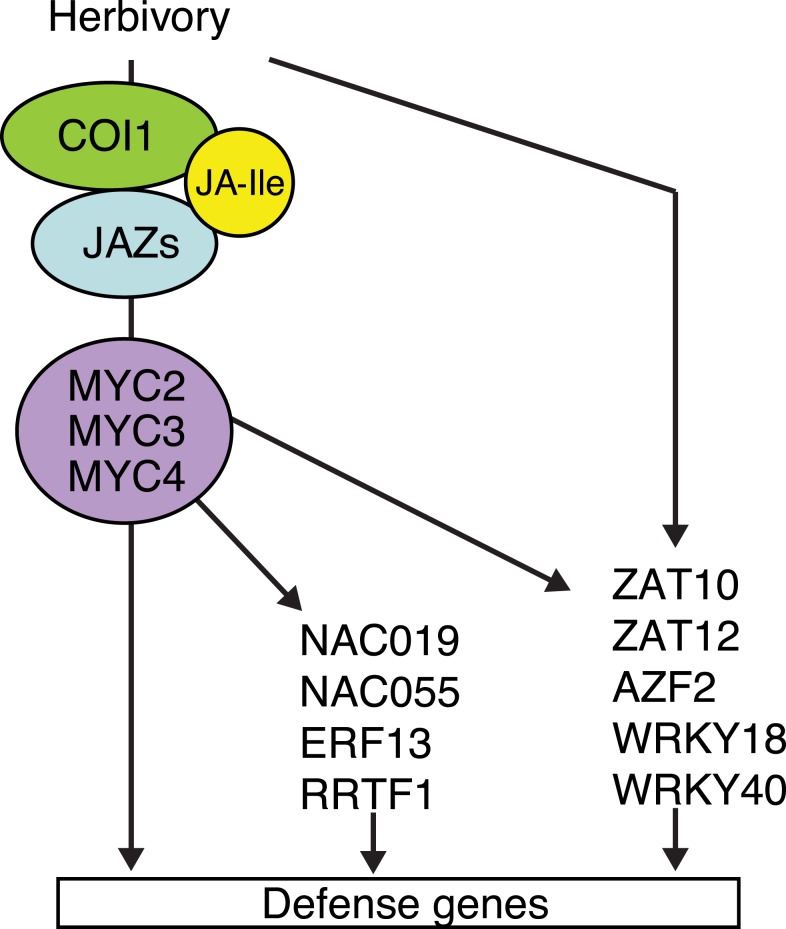
**A model for the transcriptional network in defense against chewing insects**. In response to herbivory, plants produce JA-Ile. This hormone is detected by its receptor COI1 that in turn degrades JAZ repressors (not shown) to allow the transcriptional activity of MYC2, MYC3, and MYC4 TFs. As a consequence, MYCs activate the expression defense genes and downstream TFs. In addition, herbivory induces the expression of several TFs, which partially depend on COI1 and MYCs. How these TFs are regulated and which are their target genes remains unknown. This model only contains TFs described in this study.

During insect herbivory, plants induce about 1000 genes, of which roughly 65% are regulated by the JA-pathway (Halitschke et al., 2001; Reymond et al., 2004; de Vos et al., 2005; Devoto et al., 2005). Following JA-Ile perception by COI1, repression of MYC2, MYC3, and MYC4 by JAZs is released allowing the transcription of defense genes. Consequently, *coi1-1* and *myc234* mutants display a strong susceptibility to herbivory (Fernández-Calvo et al., 2011). In order to identify novel TFs involved in plant response to herbivory, we performed a whole-genome expression analysis and found 41 TFs that were robustly induced after short- or long-term feeding by the generalist *S. littoralis*. From these, we obtained 11 mutants of which nine were found to increase insect performance. However, mutation in none of these TFs was able to phenocopy the severe susceptibility observed with *coi1-1* and *myc234*, suggesting that these factors only partially contribute to insect defense. One explanation could be that these TFs are downstream targets of MYCs and that they regulate subsets of defense genes. However, analysis of their expression in *coi1-1* and *myc234* revealed that this was not always the case. Whereas *NAC019*, *NAC055*, *ERF13*, and *RRTF1* induction by herbivory was clearly dependent on COI1 and MYC2/MYC3/MYC4, expression of other TFs was not, or only partly, affected in the mutants. We thus propose a model where groups of TFs activates defense gene expression in JA-dependent and JA-independent manner (Figure 7). For the JA-dependent pathway, MYC2/MYC3/MYC4 play a quantitatively important role by directly activating defense genes or by activating downstream TFs. In parallel, a JA-independent pathway triggers WRKYs and Zinc-finger TFs expression to provide additional defense. These findings might however represent only a fraction of all TFs involved in defense against herbivory. First, we could only obtain 11 confirmed mutants and the implication of the other insect-induced TFs should be tested. Second, it is also possible that important TFs are not induced by herbivory. For example, expression of MYC3 and MYC4 is not up-regulated by JA treatment (Fernández-Calvo et al., 2011).

Although *myc234* and *coi1-1* were equally sensitive to herbivory, confirming previous observations (Fernández-Calvo et al., 2011), we found that their expression profile was not identical and several potential defense genes were induced in Col-0 and *myc234* but not in *coi1-1*. This suggests that other yet unknown TFs are targets of the COI1-JAIle-JAZs signaling module. Since the JA-pathway is also crucial for defense against necrotrophic fungi (Thomma et al., 2000; Thaler et al., 2004), it is plausible to postulate that specific TFs are involved in this response. Recently, it was shown that JAZs bind to ethylene-stabilized TFs EIN3 and EIL1 to repress the activation of downstream genes *ERF1* and *PDF1.2* (Zhu et al., 2011). Interestingly, *PDF1.2* induction by *S. littoralis* was larger in *myc234*, *wrky18wrky40*, *zat10*, *zat12*, *nac019*, and *nac055* than in Col-0, whereas it was abolished in *coi1-1*, indicating that this branch of the JA-pathway, that requires also ethylene, might be under the negative regulation of the MYC2/MYC3/MYC4 branch. It would be interesting to test the response of *myc234* and TF mutants to necrotrophic fungi.

Global expression profiles of most TF mutants in response to herbivory displayed a moderate change compared to Col-0, whereas *coi1-1* or *myc234* had a marked reduction of defense gene expression. However, these mutants displayed a significant increased sensitivity to *S. littoralis*, indicating that each TF is controlling the expression of important defense genes. One interpretation could be that mutation in these TFs strongly affected the basal expression of defense genes and, whereas the expression ratios were similar between wild-type and mutant plants, the absolute expression level in insect-treated plants might be considerably lower. However, an analysis of expression levels of the most highly insect-induced genes did not show a drastic difference between Col-0 and TF mutants (not shown). Alternatively, the enhanced susceptibility of TF mutant plants might be due to a small but general reduction of defense gene expression. Finally, the downregulation of a few specific genes that have a strong impact on defense could also explain these results. Future research will be required to elucidate which hypothesis is true.

Results from whole-genome expression profiles placed *wrky* mutants in a distinct subgroup. Induction of the anti-insect protein VSP2 (Liu et al., 2005) was partially reduced in *wrky18*, *wrky40*, and more strongly in *wrk18wrky40* mutants. The expression of genes related to several pathways was also affected, in particular GS biosynthesis and phenylpropanoid biosynthesis were affected. Since GS are known insect deterrents, the altered GS-profile observed in *wrky* mutants might have contributed to their enhanced susceptibility. The phenylpropanoid pathway provides precursors of various secondary metabolites related to abiotic and biotic stress, including sinapate esters, lignin, suberin, flavonols, and anthocyanins (Vogt, 2010). Polyphenols include also insect repellents like catechin, rotenone, and phaseolin (Schoonhoven et al., 2005). However these compounds are not produced in *Arabidopsis* and it remains still debatable whether anthocyanins or any metabolite derived from the phenylpropanoid pathway have deterrent effects against herbivores. Our observation that genes from the last steps of anthocyanins and flavonols biosynthesis are no longer induced by *S. littoralis* in *wrky18wrky40* suggests that these compounds might be important for defense. It would thus be interesting to perform a targeted metabolic profiling of this mutant.

It was reported previously that WRKY18 and WRKY40 have opposite effects on resistance to necrotrophic and biotrophic pathogens. A *wrky18wrky40* double mutant was more susceptible to *Botrytis cinerea* while it was more resistant to *Pseudomonas syringae* (Xu et al., 2006). Since defense to *B. cinerea* requires a functional JA-pathway (Rowe et al., 2010), these WRKYs might play an important role in JA-mediated responses. In addition, a recent study found that WRKY18 and WRKY40 were involved in early ABA signaling (Shang et al., 2010). Interestingly, ABA deficient mutants have been shown to be more sensitive to herbivore insects (Bodenhausen and Reymond, 2007). A more detailed analysis of the respective roles of GS, phenylpropanoids and/or defense proteins in the WRKY-dependent response to herbivory will be interesting in the future. In addition, WRKY60, a close homolog of WRKY18, and WRKY40, was shown to form homo- and hetero-complexes with these factors and played a partially redundant role in *Arabidopsis* response to *B. cinerea* and *P. syringae* (Xu et al., 2006). A study of *wrky18/40/60* triple mutant might unveil an increased susceptibility to *S. littoralis* herbivory and a more pronounced alteration of the transcriptome.

Although there were no marked overall differences in expression patterns of *nac019*, *nac055*, and *nac019nac055* mutants compared to Col-0, these profiles were nevertheless not identical. Previously, these two TFs have been shown to be regulated by MYC2, to form homo-and hetero-dimers and to directly control the expression of *VSP1*, a close homolog of *VSP2* (Bu et al., 2008). Besides forming a distinct clade in the NAC protein family, NAC019, NAC055, and their homolog NAC072/RD26 have been shown to bind *in vitro* to the CATGTG motif (Tran et al., 2004), a G-box like motif to which MYC2, MYC3, and MYC4 also bind with high affinity (Fernández-Calvo et al., 2011). This would suggest that MYCs and NACs compete for the same binding site or form a complex. Consistent with the presence of at least two G-boxes in the promoter of *NAC019* and *NAC055*, we found that their induction by herbivory was highly reduced in *myc234*, which suggests that MYC2, MYC3, and MYC4 directly regulate the expression of these genes. Indeed, a recent study showed that MYC2 binds directly to the promoter of *NAC019*, *NAC055*, and *NAC072* and that these TFs positively regulate coronatine-mediated suppression of the salicylic acid (SA) pathway (Zheng et al., 2012). The negative cross-talk between JA and SA is a relatively well-understood process (Pieterse et al., 2012) which could explain the insect sensitive phenotypes of *nac* mutants. In the presence of insects, JA might represses the SA signaling pathway via these NAC TFs, whereas elevated SA, as observed in triple *nac* mutants, repress the JA signaling pathway (Zheng et al., 2012). The fact that whole-genome transcription analysis did not show any major differences between *nac019*, *nac055*, *nac019nac055*, and Col-0 in response to herbivory might have been due to a compensatory effect of NAC072 and may have thus prevented a comprehensive characterization of the role of NACs in insect defense. Further investigations with *nac019/055/072* triple mutants will be needed to determine whether NACs directly regulate insect defense genes or whether this is done indirectly by repressing the SA pathway.

ZAT12 and ZAT10 are known to play a role in plant defense to oxidative stress. It was reported that *zat12* plants are more sensitive to H_2_O_2_ application and are unable to activate ROS-scavenging transcripts (Rizhsky et al., 2004). In addition, overexpression of ZAT10 elevated the expression of ROS-responsive genes (Mittler et al., 2006). Since ROS production has been implicated in defense against herbivores (Kerchev et al., 2012), the enhanced susceptibility of *zat10* and *zat12* mutants could be explained by a decreased ability to generate ROS. Further experiments will be required to test this hypothesis.

Finally, *erf13* and *rrtf1*, two TF mutants that belong to the same sub-family of ERF/AP2 factors, were clustered separately from Col-0 and other TF mutants. This difference can be attributed to the fact that both mutants had a more pronounced induction of many insect-induced genes. However, since these mutants were more susceptible to *S. littoralis* herbivory, it does not seem that this enhanced expression played a significant role in their response to herbivory.

In conclusion, this study demonstrates the involvement of several novel TFs in plant defense against insects. We find that MYC2/MYC3/MYC4 are the main contributors of resistance to a generalist herbivore and that they constitute a central hub that controls the expression of downstream TFs. In addition, JA-independent factors also contribute significantly to defense. In the future, more work will be necessary to identify the complete regulatory network and associated genes that are involved in defense against insects.

## Conflict of Interest Statement

The authors declare that the research was conducted in the absence of any commercial or financial relationships that could be construed as a potential conflict of interest.

## Supplementary Material

The Supplementary Material for this article can be found online at http://www.frontiersin.org/Plant-Microbe_Interaction/10.3389/fpls.2013.00013/abstract

Supplementary Table S1 **List of insect-induced TFs**.

Supplementary Table S2 **Whole-genome expression data for Col-0 and TF mutants**.

Supplementary Table S3 **List of primers used in this study**.

## Footnotes

^1^http://signal.salk.edu/tdnaprimers.2.html

^2^http://www.tm4.org/mev/

^3^http://www.ebi.ac.uk/arrayexpress/

^4^www.arabidopsis.org

## References

[B1] Baldwin I. T. (1998). Jasmonate-induced responses are costly but benefit plants under attack in native populations. Proc. Natl. Acad. Sci. U.S.A. 95, 8113–8118 10.1073/pnas.95.14.8113 9653149 PMC20938

[B2] Beekwilder J. van Leeuwen W. van Dam N. M. Bertossi M. Grandi V. Mizzi L. (2008). The impact of the absence of aliphatic glucosinolates on insect herbivory in *Arabidopsis*. PLoS ONE 3:e2068 10.1371/journal.pone.0002068 18446225 PMC2323576

[B3] Bodenhausen N. Reymond P. (2007). Signaling pathways controlling induced resistance to insect herbivores in *Arabidopsis*. Mol. Plant Microbe Interact. 20, 1406–1420 10.1094/MPMI-20-11-1406 17977152

[B4] Boter M. Ruíz-Rivero O. Abdeen A. Prat S. (2004). Conserved MYC transcription factors play a key role in jasmonate signaling both in tomato and *Arabidopsis*. Genes Dev. 18, 1577–1591 10.1101/gad.297704 15231736 PMC443520

[B5] Bu Q. Jiang H. Li C.-B. Zhai Q. Zhang J. Wu X. (2008). Role of the *Arabidopsis thaliana* NAC transcription factors ANAC019 and ANAC055 in regulating jasmonic acid-signaled defense responses. Cell Res. 18, 756–767 10.1038/cr.2008.53 18427573

[B6] Chini A. Fonseca S. Fernández G. Adie B. A. T. Chico J. M. Lorenzo O. (2007). The JAZ family of repressors is the missing link in jasmonate signalling. Nature 448, 666–671 10.1038/nature06006 17637675

[B7] de Vos M. Denekamp M. Dicke M. Vuylsteke M. Van Loon L. C. Smeekens S. C. (2006). The *Arabidopsis thaliana* transcription factor AtMYB102 functions in defense against the insect herbivore *Pieris rapae*. Plant Signal. Behav. 1, 305–311 10.4161/psb.1.6.3512 19517001 PMC2634245

[B8] de Vos M. Van Oosten V. R. Van Poecke R. M. P. Van Pelt J. A. Pozo M. J. Mueller M. J. (2005). Signal signature and transcriptome changes of *Arabidopsis* during pathogen and insect attack. Mol. Plant Microbe Interact. 18, 923–937 10.1094/MPMI-18-0923 16167763

[B9] Devoto A. Ellis C. Magusin A. Chang H.-S. Chilcott C. Zhu T. (2005). Expression profiling reveals COI1 to be a key regulator of genes involved in wound- and methyl jasmonate-induced secondary metabolism, defence, and hormone interactions. Plant Mol. Biol. 58, 497–513 10.1007/s11103-005-7306-5 16021335

[B10] Dombrecht B. Xue G. P. Sprague S. J. Kirkegaard J. A. Ross J. J. Reid J. B. (2007). MYC2 differentially modulates diverse jasmonate-dependent functions in *Arabidopsis*. Plant Cell 19, 2225–2245 10.1105/tpc.106.048017 17616737 PMC1955694

[B11] Fernández-Calvo P. Chini A. Fernández-Barbero G. Chico J. M. Gimenez-Ibanez S. Geerinck J. (2011). The *Arabidopsis* bHLH transcription factors MYC3 and MYC4 are targets of JAZ repressors and act additively with MYC2 in the activation of jasmonate responses. Plant Cell 23, 701–715 10.1105/tpc.110.080788 21335373 PMC3077776

[B12] Feys B. J. Benedetti C. E. Penfold C. N. Turner J. G. (1994). *Arabidopsis* mutants selected for resistance to the phytotoxin coronatine are male sterile, insensitive to methyl jasmonate, and resistant to a bacterial pathogen. Plant Cell 6, 751–759 10.1105/tpc.6.5.751 12244256 PMC160473

[B13] Fonseca S. Chini A. Hamberg M. Adie B. Porzel A. Kramell R. (2009). (+)-7-iso-Jasmonoyl-L-isoleucine is the endogenous bioactive jasmonate. Nat. Chem. Biol. 5, 344–350 10.1038/nchembio.161 19349968

[B14] Gigolashvili T. Berger B. Mock H.-P. Müller C. Weisshaar B. Flügge U.-I. (2007). The transcription factor HIG1/MYB51 regulates indolic glucosinolate biosynthesis in *Arabidopsis thaliana*. Plant J. 50, 886–901 10.1111/j.1365-313X.2007.03099.x 17461791

[B15] Gigolashvili T. Engqvist M. Yatusevich R. Müller C. Flügge U.-I. (2008). HAG2/MYB76 and HAG3/MYB29 exert a specific and coordinated control on the regulation of aliphatic glucosinolate biosynthesis in *Arabidopsis thaliana*. New Phytol. 177, 627–642 10.1111/j.1469-8137.2007.02295.x 18042203

[B16] Glauser G. Schweizer F. Turlings T. C. J. Reymond P. (2012). Rapid profiling of intact glucosinolates in *Arabidopsis* leaves by UHPLC-QTOFMS using a charged surface hybrid column. Phytochem. Anal. 23, 520–528 10.1002/pca.2350 22323091

[B17] Godoy M. Franco-Zorrilla J. M. Pérez-Pérez J. Oliveros J. C. Lorenzo O. Solano R. (2011). Improved protein-binding microarrays for the identification of DNA-binding specificities of transcription factors. Plant J. 66, 700–711 10.1111/j.1365-313X.2011.04519.x 21284757

[B18] Grubb C. Abel S. (2006). Glucosinolate metabolism and its control. Trends Plant Sci. 11, 89–100 10.5363/tits.11.9_89 16406306

[B19] Halitschke R. Schittko U. Pohnert G. Boland W. Baldwin I. T. (2001). Molecular interactions between the specialist herbivore *Manduca sexta* (Lepidoptera, Sphingidae) and its natural host *Nicotiana attenuata*. III. Fatty acid-amino acid conjugates in herbivore oral secretions are necessary and sufficient for herbivore-specific plant responses. Plant Physiol. 125, 711–717 10.1104/pp.125.2.711 11161028 PMC64872

[B20] Halkier B. A. Gershenzon J. (2006). Biology and biochemistry of glucosinolates. Annu. Rev. Plant Biol. 57, 303–333 10.1146/annurev.arplant.57.032905.105228 16669764

[B21] Hirai M. Y. Sugiyama K. Sawada Y. Tohge T. Obayashi T. Suzuki A. (2007). Omics-based identification of *Arabidopsis* Myb transcription factors regulating aliphatic glucosinolate biosynthesis. Proc. Natl. Acad. Sci. U.S.A. 104, 6478–6483 10.1073/pnas.0611629104 17420480 PMC1849962

[B22] Howe G. A. Jander G. (2007). Plant immunity to insect herbivores. Annu. Rev. Plant Biol. 59, 41–66 10.1146/annurev.arplant.59.032607.092825 18031220

[B23] Howe G. A. Lightner J. Browse J. Ryan C. A. (1996). An octadecanoid pathway mutant (JL5) of tomato is compromised in signaling for defense against insect attack. Plant Cell 8, 2067–2077 10.2307/3870413 8953771 PMC161335

[B24] Johnson E. T. Dowd P. F. (2004). Differentially enhanced insect resistance, at a cost, in *Arabidopsis thaliana* constitutively expressing a transcription factor of defensive metabolites. J. Agric. Food Chem. 52, 5135–5138 10.1021/jf0308049 15291486

[B25] Kerchev P. I. Fenton B. Foyer C. H. Hancock R. D. (2012). Plant responses to insect herbivory: interactions between photosynthesis, reactive oxygen species and hormonal signalling pathways. Plant Cell Environ. 35, 441–453 10.1111/j.1365-3040.2011.02395.x 21752032

[B26] Li L. Zhao Y. McCaig B. C. Wingerd B. A. Wang J. Whalon M. E. (2004). The tomato homolog of CORONATINE-INSENSITIVE1 is required for the maternal control of seed maturation, jasmonate-signaled defense responses, and glandular trichome development. Plant Cell 16, 126–143 10.1105/tpc.017954 14688297 PMC301400

[B27] Liechti R. Csárdi G. Bergmann S. Schütz F. Sengstag T. Boj S. F. (2010). EuroDia: a beta-cell gene expression resource. Database (Oxford) 2010, baq024 10.1093/database/baq004 20940178 PMC2963318

[B28] Liu Y. Ahn J.-E. Datta S. Salzman R. A. Moon J. Huyghues-Despointes B. (2005). *Arabidopsis* vegetative storage protein is an anti-insect acid phosphatase. Plant Physiol. 139, 1545–1556 10.1104/pp.105.063420 16258019 PMC1283788

[B29] Lorenzo O. Chico J. M. Sánchez-Serrano J. J. Solano R. (2004). JASMONATE-INSENSITIVE1 encodes a MYC transcription factor essential to discriminate between different jasmonate-regulated defense responses in *Arabidopsis*. Plant Cell 16, 1938–1950 10.1105/tpc.022319 15208388 PMC514172

[B30] Manners J. M. J. Penninckx I. A. I. Vermaere K. K. Kazan K. K. Brown R. L. R. Morgan A. A. (1998). The promoter of the plant defensin gene PDF1.2 from *Arabidopsis* is systemically activated by fungal pathogens and responds to methyl jasmonate but not to salicylic acid. Plant Mol. Biol. 38, 1071–1080 10.1023/A:1006070413843 9869413

[B31] McConn M. Creelman R. A. Bell E. Mullet J. E. Browse J. (1997). Jasmonate is essential for insect defense in *Arabidopsis*. Proc. Natl. Acad. Sci. U.S.A. 94, 5473–5477 10.1073/pnas.94.10.5473 11038546 PMC24703

[B32] Mewis I. Tokuhisa J. G. Schultz J. C. Appel H. M. Ulrichs C. Gershenzon J. (2006). Gene expression and glucosinolate accumulation in *Arabidopsis thaliana* in response to generalist and specialist herbivores of different feeding guilds and the role of defense signaling pathways. Phytochemistry 67, 2450–2462 10.1016/j.phytochem.2006.09.004 17049571

[B33] Misra P. Pandey A. Tiwari M. Chandrashekar K. Sidhu O. P. Asif M. H. (2010). Modulation of transcriptome and metabolome of tobacco by *Arabidopsis* transcription factor, AtMYB12, leads to insect resistance. Plant Physiol. 152, 2258–2268 10.1104/pp.109.150979 20190095 PMC2850017

[B34] Mittler R. Kim Y. Song L. Coutu J. Coutu A. Ciftci-Yilmaz S. (2006). Gain- and loss-of-function mutations in Zat10 enhance the tolerance of plants to abiotic stress. FEBS Lett. 580, 6537–6542 10.1016/j.febslet.2006.11.002 17112521 PMC1773020

[B35] Paschold A. Halitschke R. Baldwin I. T. (2007). Co(i)-ordinating defenses: NaCOI1 mediates herbivore- induced resistance in *Nicotiana attenuata* and reveals the role of herbivore movement in avoiding defenses. Plant J. 51, 79–91 10.1111/j.1365-313X.2007.03119.x 17561925

[B36] Pauwels L. Barbero G. F. Geerinck J. Tilleman S. Grunewald W. Pérez A. C. (2010). NINJA connects the co-repressor TOPLESS to jasmonate signalling. Nature 464, 788–791 10.1038/nature08854 20360743 PMC2849182

[B37] Pauwels L. Goossens A. (2011). The JAZ proteins: a crucial interface in the jasmonate signaling cascade. Plant Cell 23, 3089–3100 10.1105/tpc.111.089300 21963667 PMC3203442

[B38] Penninckx I. A. Thomma B. P. H. J. Buchala A. J. Métraux J. P. Broekaert W. F. (1998). Concomitant activation of jasmonate and ethylene response pathways is required for induction of a plant defensin gene in *Arabidopsis*. Plant Cell 10, 2103–2113 10.2307/3870787 9836748 PMC143966

[B39] Pieterse C. M. J. der Does Van D. Zamioudis C. Leon-Reyes A. Van Wees S. C. M. (2012). Hormonal modulation of plant immunity. Annu. Rev. Cell Dev. Biol. 28, 489–521 10.1146/annurev-cellbio-092910-154055 22559264

[B40] Pré M. M. Atallah M. M. Champion A. A. De Vos M. M. Pieterse C. M. J. C. Memelink J. J. (2008). The AP2/ERF domain transcription factor ORA59 integrates jasmonic acid and ethylene signals in plant defense. Plant Physiol. 147, 1347–1357 10.1104/pp.108.117523 18467450 PMC2442530

[B41] Reymond P. Bodenhausen N. Van Poecke R. M. P. Krishnamurthy V. Dicke M. Farmer E. E. (2004). A conserved transcript pattern in response to a specialist and a generalist herbivore. Plant Cell 16, 3132–3147 10.1105/tpc.104.026120 15494554 PMC527203

[B42] Reymond P. Weber H. Damond M. Farmer E. E. (2000). Differential gene expression in response to mechanical wounding and insect feeding in *Arabidopsis*. Plant Cell 12, 707–720 10.1105/tpc.12.5.707 10810145 PMC139922

[B43] Rizhsky L. Davletova S. Liang H. Mittler R. (2004). The zinc finger protein Zat12 is required for cytosolic ascorbate peroxidase 1 expression during oxidative stress in *Arabidopsis*. J. Biol. Chem. 279, 11736–11743 10.1074/jbc.M313350200 14722088

[B44] Rowe H. C. Walley J. W. Corwin J. Chan E. K.-F. Dehesh K. Kliebenstein D. J. (2010). Deficiencies in jasmonate-mediated plant defense reveal quantitative variation in *Botrytis cinerea* pathogenesis. PLoS Pathog. 6:e1000861 10.1371/journal.ppat.1000861 20419157 PMC2855333

[B45] Schoonhoven L. M. van Loon J. J. A. Dicke M. (2005). Insect-Plant Biology. Oxford: Oxford University Press

[B46] Sclep G. Allemeersch J. Liechti R. De Meyer B. Beynon J. Bhalerao R. (2007). CATMA, a comprehensive genome-scale resource for silencing and transcript profiling of *Arabidopsis* genes. BMC Bioinformatics 8:400 10.1186/1471-2105-8-400 17945016 PMC2147040

[B47] Shang Y. Yan L. Liu Z.-Q. Cao Z. Mei C. Xin Q. (2010). The Mg-chelatase H subunit of *Arabidopsis* antagonizes a group of WRKY transcription repressors to relieve ABA-responsive genes of inhibition. Plant Cell 22, 1909–1935 10.1105/tpc.110.073874 20543028 PMC2910980

[B48] Sheard L. B. Tan X. Mao H. Withers J. Ben-Nissan G. Hinds T. R. (2010). Jasmonate perception by inositol-phosphate-potentiated COI1-JAZ co-receptor. Nature 468, 400–405 10.1038/nature09430 20927106 PMC2988090

[B49] Skibbe M. Qu N. Galis I. Baldwin I. T. (2008). Induced plant defenses in the natural environment: *Nicotiana attenuata* WRKY3 and WRKY6 coordinate responses to herbivory. Plant Cell 20, 1984–2000 10.1105/tpc.108.058594 18641266 PMC2518244

[B50] Sønderby I. E. Burow M. Rowe H. C. Kliebenstein D. J. Halkier B. A. (2010). A complex interplay of three R2R3 MYB transcription factors determines the profile of aliphatic glucosinolates in *Arabidopsis*. Plant Physiol. 153, 348–363 10.1104/pp.109.149286 20348214 PMC2862430

[B51] Stintzi A. Weber H. Reymond P. Browse J. Farmer E. E. (2001). Plant defense in the absence of jasmonic acid: the role of cyclopentenones. Proc. Natl. Acad. Sci. U.S.A. 98, 12837–12842 10.1073/pnas.211311098 11592974 PMC60140

[B52] Thaler J. S. Owen B. Higgins V. J. (2004). The role of the jasmonate response in plant susceptibility to diverse pathogens with a range of lifestyles. Plant Physiol. 135, 530–538 10.1104/pp.104.041566 15133157 PMC429405

[B53] Thines B. Katsir L. Melotto M. Niu Y. Mandaokar A. Liu G. (2007). JAZ repressor proteins are targets of the SCF(COI1) complex during jasmonate signalling. Nature 448, 661–665 10.1038/nature05960 17637677

[B54] Thomma B. P. H. J. Eggermont K. Broekaert W. F. (2000). Disease development of several fungi on *Arabidopsis* can be reduced by treatment with methyl jasmonate. Plant Physiol. Biochem. 38, 421–427 10.1016/S0981-9428(00)00756-7

[B55] Tran L.-S. P. Nakashima K. Sakuma Y. Simpson S. D. Fujita Y. Maruyama K. (2004). Isolation and functional analysis of *Arabidopsis* stress-inducible NAC transcription factors that bind to a drought-responsive cis-element in the early responsive to dehydration stress 1 promoter. Plant Cell 16, 2481–2498 10.1105/tpc.104.022699 15319476 PMC520947

[B56] Verhage A. Vlaardingerbroek I. Raaijmakers C. Van Dam N. Dicke M. Van Wees S. C. M. (2011). Rewiring of the jasmonate signaling pathway in *Arabidopsis* during insect gerbivory. Front. Plant Sci. 2:47 10.3389/fpls.2011.00047 22645537 PMC3355780

[B57] Vogt T. (2010). Phenylpropanoid biosynthesis. Mol. Plant 3, 2–20 10.1093/mp/ssp106 20035037

[B58] Walling L. L. (2000). The myriad plant responses to herbivores. J. Plant Growth Regul. 19, 195–216 11038228 10.1007/s003440000026

[B59] Wittstock U. Gershenzon J. (2002). Constitutive plant toxins and their role in defense against herbivores and pathogens. Curr. Opin. Plant Biol. 5, 300–307 10.1016/S1369-5266(02)00264-9 12179963

[B60] Xie D. Feys B. J. James S. Nieto-Rostro M. Turner J. G. (1998). COI1: an *Arabidopsis* gene required for jasmonate-regulated defense and fertility. Science 280, 1091–1094 10.1126/science.280.5362.443 9582125

[B61] Xu X. Chen C. Fan B. Chen Z. (2006). Physical and functional interactions between pathogen-induced *Arabidopsis* WRKY18, WRKY40, and WRKY60 transcription factors. Plant Cell 18, 1310–1326 10.1105/tpc.105.037523 16603654 PMC1456877

[B62] Yan Y. Stolz S. Chételat A. Reymond P. Pagni M. Dubugnon L. (2007). A downstream mediator in the growth repression limb of the jasmonate pathway. Plant Cell 19, 2470–2483 10.1105/tpc.107.050708 17675405 PMC2002611

[B63] Zheng X.-Y. Spivey N. W. Zeng W. Liu P.-P. Fu Z. Q. Klessig D. F. (2012). Coronatine promotes *Pseudomonas syringae* virulence in plants by activating a signaling cascade that inhibits salicylic acid accumulation. Cell Host Microbe 11, 587–596 10.1016/j.chom.2012.04.014 22704619 PMC3404825

[B64] Zhu Z. An F. Feng Y. Li P. Xue L. A. M. Jiang Z. (2011). Derepression of ethylene-stabilized transcription factors (EIN3/EIL1) mediates jasmonate and ethylene signaling synergy in *Arabidopsis*. Proc. Natl. Acad. Sci. U.S.A. 108, 12539–12544 10.1073/pnas.1103959108 21737749 PMC3145709

