# Supplementary material for: Differential Contribution of Transcription Factors to Arabidopsis thaliana Defense Against Spodoptera littoralis
Source: Front Plant Sci. 2013 Feb 4;4:13. doi: 10.3389/fpls.2013.00013 (PMC3563046; doi:10.3389/fpls.2013.00013)
Supplement: Supplementary Table S3 — List of primers used in this study. [file 42585_Reymond_DataSheet3.DOCX]

**Table S3.** List of primers used in this study

AGI Gene Primer Sequence (5' - 3')

List of primers used for T-DNA insertion verification

AT1G52890 NAC019 SALK_096295_Fw CGACGATCTAACTGACACACG

SALK_096295_Rv ATGCGGTTTGGGTTAGAAAAC

AT3G15500 NAC055 SALK_014331_Fw TAAACGATGAGCGATAGCGAG

SALK_014331_Rv AAAGGAACCAAAACCAATTGG

AT1G27730 ZAT10 SALK_054092_Fw TTACCAACACCACTTGCACAC

SALK_054092_Rv AAGTCAAACCGAGGCTTCTTC

AT5G59820 ZAT12 SAIL_347_G03_Fw CAGGAGAACCAATGAGCTCTG

SAIL_347_G03_Rv TGCGATATCGGAGATCAAGTC

AT3G19580 AZF2 SALK_132562_Fw TTGTTGCTAACAAGCATGTGC

SALK_132562_Rv GCCAGAATCAAAGAACCTTCC

AT4G31800 WRKY18 SALK_093916_Fw CGACAGTAAATTTAACCCATGC

SALK_093916_Rv CAAAGACATTCAAACTCAGACCC

AT4G34410 RRTF1 SALK_150614_Fw CGCGATGCTTTGTAGGAGTAG

SALK_150614_Rv GATCTCAGGGGAAAACGAAAC

AT2G44840 ERF13 GK_121A12_Fw CAAAATATCAAAGAACAGCCAAC

GK_121A12_Rv CGAAGTAAAACTGATCCACCG

AT1G43160 RAP2.6 SAIL_1225_G09_Fw TCAATCAACGTGTCATGAAGG

SAIL_1225_G09_Rv TCAGACTGAAGTTGTATTGGGAG

AT5G67300 MYB44 SALK_039074_Fw AAATTCACCTCAAATTGTGCG

SALK_039074_Rv CACAGGCTTGAAAAGCTCAAC

List of primers used for qPCR analysis

AT5G44420 PDF1.2 PDF1.2_Fw AGTTGTGCGAGAAGCCAAGT

PDF1.2_Rv GTTGCATGATCCATGTTTGG

AT5G24770 VSP2 VSP2_Fw CGTCGATTCGAAAACCATCT

VSP2_Rv GGCACCGTGTCGAAGTCTAT

AT4G34410 RRTF1 RRTF1_Fw TCATCTCCTGTTGCTGCTGA

RRTF1_Rv CATATTGCAATCCCCTCCTC

AT2G44840 ERF13 ERF13_Fw CCGTCAGTCTCCGATCAGTT

ERF13_Rv GATCCACCGTGAAATCCAAC

AT4G31800 WRKY18 WRKY18_Fw CAAGAGACAACCCGTCACCT

WRKY18_Rv CGCTGCGTTGTACCTTCTTT

AT1G80840 WRKY40 WRKY40_Fw GAGACAATCCATCTCCAAGAGC

WRKY40_Rv CGGACTGATCCTCCACACTT

AT3G19580 AZF2 AZF2_Fw CACGGTATCGGAAGAAAGGA

AZF2_Rw TTTTACCGGTCAACGGACTC

AT1G52890 NAC019 NAC019_Fw CAAATTCGATCCATGGGTTT

NAC019_Rv TTAGGTCTTGACCCGTTTGG

AT3G15500 NAC055 NAC055_Fw TCTCATCGAACCCTCTCGTC

NAC055_Rv TCATGCAACGACTCGAGAAC

AT1G27730 ZAT10 ZAT10_Fw GGTCCACTAGCCACGTTAGC

ZAT10_Rv TTTGACCGGAAAGTCAAACC

AT5G59820 ZAT12 ZAT12_Fw ATCAAGTCGACGGTGGATGT

ZAT12_Rv CCTAAGGCTTGGAACGAATG

AT5G54060 3GT 3GT_Fw TTGATGGCAAGGAAATGTCA

3GT_Rv CCACACGAAACTCAGGGATT

AT5G42800 DFR DFR_Fw CCAAATTTCTCAGGCCAAAA

DFR_Rv TCCGTCAGCTTCTTGGAACT

AT4G22880 LDOX LDOX_Fw TGGGTCACTGCAAAATGTGT

LDOX_Rv TATTCACCAACCCACGATGA
